# "White-Out Lung" Following Veno-Venous Extracorporeal Membrane Oxygenation (ECMO) Initiation for Acute Refractory Respiratory Failure From Obstructive Lung Disease: A Case Series

**DOI:** 10.7759/cureus.116514

**Published:** 2026-09-18

**Authors:** Santhalakshmi Angappan, Joseph Brisson, Nakul S Kumar

**Affiliations:** 1 Anesthesiology and Critical Care, Cleveland Clinic Foundation, Cleveland, USA

**Keywords:** complications of ecmo, lung opacity in ecmo, ventilator management, vv-ecmo for refractory asthma and copd, white-out lung in vv-ecmo

## Abstract

Veno-venous extracorporeal membrane oxygenation (VV-ECMO) has been used as a salvage therapy for respiratory failure despite mechanical ventilation. However, evidence for its utility and associated complications remains limited.

We hereby present a case report of sudden lung collapse in patients with obstructive physiologies after initiating VV-ECMO. Our case report focuses on acute lung opacification that occurred within hours of initiating VV-ECMO. Given the growing number of ECMO runs for asthma and chronic obstructive pulmonary disease (COPD), intensive care teams should be aware of ventilatory changes and complications associated with the lung rest strategy while on VV-ECMO, facilitating early recognition and prompt intervention.

## Introduction

Veno-venous extracorporeal membrane oxygenation (VV-ECMO) has been increasingly used as a salvage therapy for acute hypoxic and hypercapnic respiratory failure despite mechanical ventilation [1,2]. Many studies have evaluated the utility of VV-ECMO in severe acute respiratory distress syndrome (ARDS); however, there is a lack of evidence for the utility and the ensuing complications of VV-ECMO for near-fatal asthma or chronic obstructive pulmonary disease (COPD) exacerbation [3]. Among patients with very severe ARDS, 60-day mortality was not significantly lower with ECMO than with a strategy of conventional mechanical ventilation that included ECMO as rescue therapy [4]. While lung rest strategies such as ultra-lung-protective ventilation (ULPV) in ECMO effectively decrease volume- and pressure-associated trauma, these shallow tidal volumes and driving pressures can predispose to significant acute atelectasis-associated trauma, leading to small airway inflammation [5]. Overall, there is a lack of evidence and literature on unilateral lung opacification following VV-ECMO initiation for acute refractory respiratory failure from obstructive lung disease, especially considering restrictive airway disease tends to cause hyperinflation. Given the growing number of ECMO runs for near-fatal asthma and COPD exacerbation in the country, it is prudent that the managing intensive care teams are familiar with the various ventilation management strategies and their associated complications for patients on ECMO for these pathologies. We hereby present a case report of three cases of sudden lung collapse in patients with similar obstructive physiologies after initiation of VV-ECMO and their management. 

## Case presentation

Case 1

A 65-year-old female with a BMI of 35 presented with a severe COPD exacerbation following severe air pollution caused by the recent Canadian wildfires. Due to refractory hypercapnic respiratory failure with high peak airway pressures on mechanical ventilation, the patient was placed on peripheral VV-ECMO. A ULPV strategy for “lung rest” was employed soon after the initiation of ECMO. The patient was on pressure-controlled continuous mandatory ventilation (PC-CMV) mode on 100% FiO₂ with a pressure control of 10 cm H₂O, positive end-expiratory pressure (PEEP) of 5, and a respiratory rate of 4/min, reducing peak airway pressures to <25 cm H₂O. The patient’s driving pressure was 20, reaching a tidal volume of 240 mL. Eventually, the patient developed unilateral collapse appearing as a “white-out” of the left lung on chest X-ray, with refractory bronchospasms despite ECMO support. Bronchoscopy was performed to rule out mucus plugging. After performing aggressive recruitment maneuvers by increasing PEEP to 14, driving pressure to 22, and respiratory rate to 10, reducing FiO₂ to 70%, and administering multidrug therapy for bronchodilation, the left lung was recruited completely. The patient was decannulated from ECMO on day 10 and transferred out of the ICU (Figures 1a-1c).

**Figure 1:**
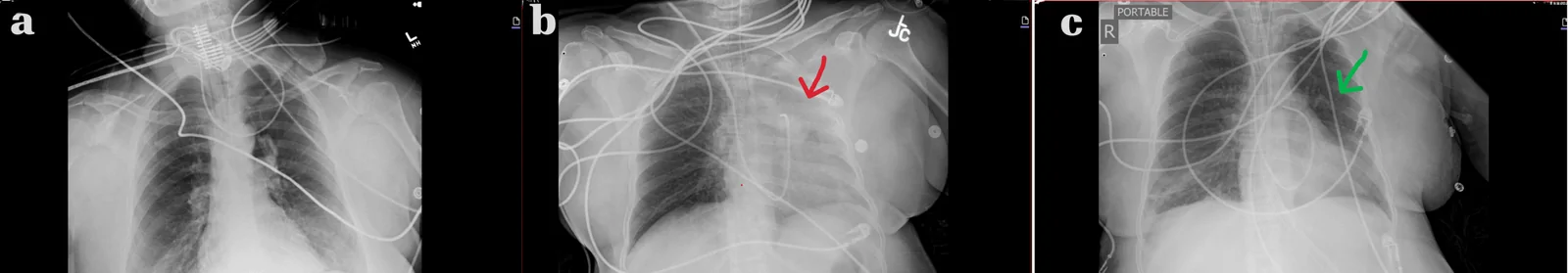
a) CXR before initiation of ECMO; b) CXR after initiation of ECMO and the lung rest strategy, with the red arrow showing left lung opacity; c) CXR after changes in ventilator settings, with the green arrow showing resolution of the left lung opacity. CXR: chest X-ray; ECMO: extracorporeal membrane oxygenation

Case 2

A 23-year-old female with a BMI of 40 and a past medical history of severe asthma exacerbations presented in respiratory distress exacerbated by noncompliance with medications. The patient was on mechanical ventilation for refractory bronchospasm and had to be initiated on VV-ECMO for refractory hypoxic-hypercarbic respiratory failure. While on ECMO, the patient was on PC-CMV mode on 100% FiO₂, with a pressure control of 12 cm H₂O, PEEP of 8, a respiratory rate of 6/min, and a driving pressure of 16. The patient developed lung opacification on chest X-ray, progressing to white-out lung. The patient's oxygenation and chest infiltrates improved significantly after aggressive recruitment maneuvers, increasing PEEP to 16, respiratory rate to 12, and driving pressure to 20 while on PC-CMV. Within a week, the patient was decannulated and extubated. She was eventually transferred out of the ICU (Figures 2a-2c). 

**Figure 2:**
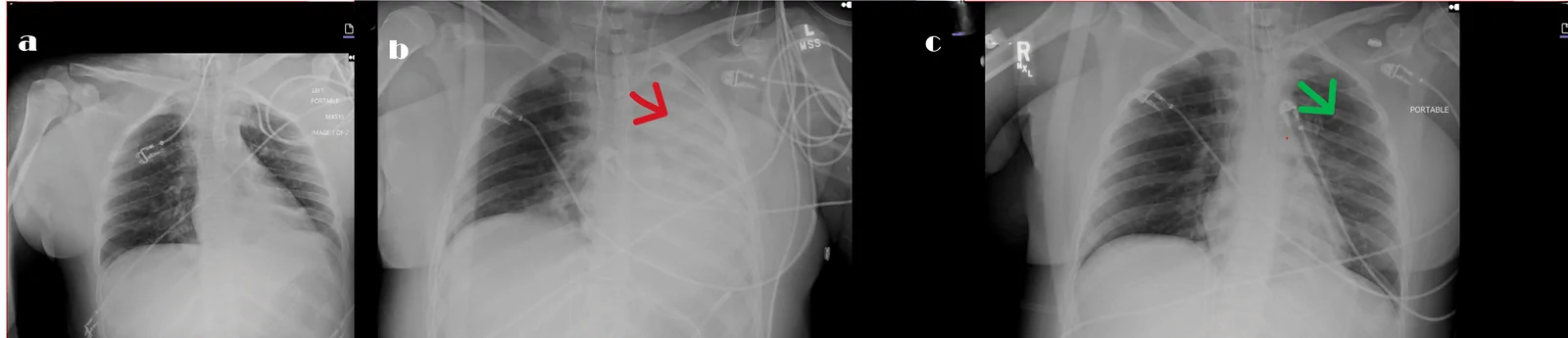
a) CXR before initiation of ECMO; b) CXR after initiation of ECMO and the lung rest strategy, with the red arrow showing left lung opacity; c) CXR after changes in ventilator settings, with the green arrow showing resolution of the left lung opacity. CXR: chest X-ray; ECMO: extracorporeal membrane oxygenation

Case 3

A 27-year-old female with a BMI of 32 presented with a severe asthma exacerbation following exposure to a family member with croup. The patient was on mechanical ventilation for refractory bronchospasm and had to be initiated on VV-ECMO for refractory hypoxic-hypercarbic respiratory failure.

While on ECMO, the patient was on PC-CMV mode on 50% FiO₂, with a pressure control of 10 cm H₂O, PEEP of 8, and a respiratory rate of 10/min. On chest X-ray, the patient developed bilateral lung opacification, which progressed to white-out lung. The patient’s oxygenation and chest infiltrates improved after increasing the respiratory rate and PEEP to 12 on PC-CMV mode. The patient was decannulated and extubated in four days. She was eventually transferred to a regular medicine floor (Figures 3a-3c).

**Figure 3:**
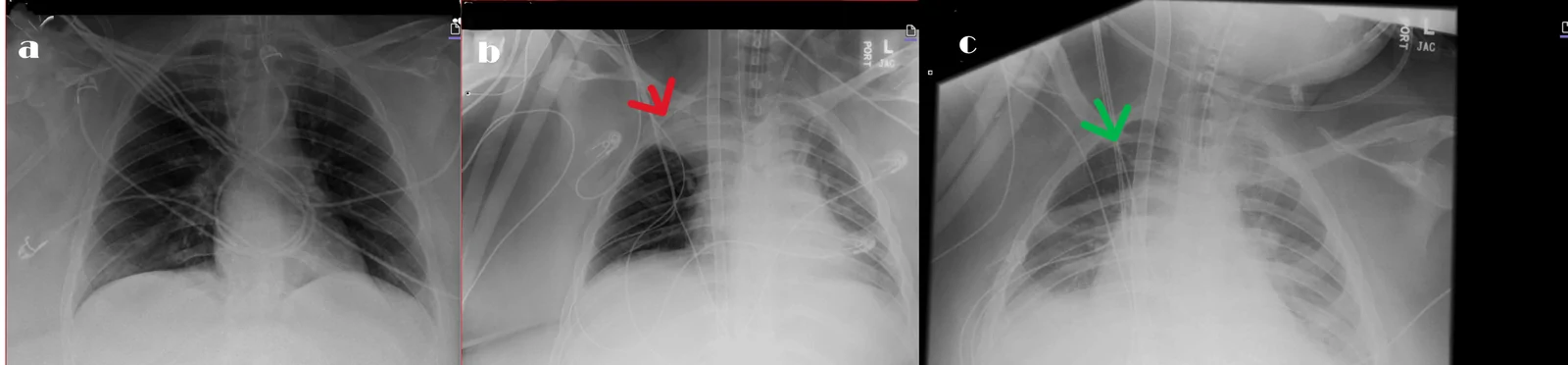
a) CXR before initiation of ECMO; b) CXR after initiation of ECMO and the lung rest strategy, with the red arrow showing right lung opacity; c) CXR after changes in ventilator settings, with the green arrow showing resolution of lung opacity. CXR: chest X-ray; ECMO: extracorporeal membrane oxygenation

## Discussion

The mechanism of hypoxia and hypercapnia in acute asthma or COPD exacerbation is ventilation-perfusion mismatch, shunting, and hypoventilation [6]. ECMO has traditionally been used to treat respiratory failure from acute lung injury (ALI) or ARDS that is refractory to conservative ventilatory management. The utilization of ECMO for severe refractory respiratory failure from near-fatal asthma or COPD exacerbation is not uncommon. In this subgroup of patients, ECMO facilitates lung rest and recovery until the inflammation causing bronchospasm subsides. The success of ECMO in obstructive lung diseases is due to the natural reversibility of the airflow obstruction. Lung-protective ventilation, inherent to ECMO, stabilizes dynamic hyperinflation in these obstructive lung diseases [7].

Recently, ventilatory management of patients on VV-ECMO has shifted toward “lung rest,” or ULPV. Ultra-protective ventilation is described as low TV (<4 mL/kg), low plateau pressures (<25 cm H₂O), low driving pressures (<14 cm H₂O), and PEEP optimization, with or without a decrease in respiratory rate [8]. “Lung rest” couples low-tidal-volume ventilation with decreased PEEP and mean airway pressure (<25 cm H₂O) [9].

However, while on a lung rest strategy, complete or partial lung collapse usually happens within hours of initiation of ECMO from alveolar edema and atelectasis because of the acute transition from maximal ventilatory support to a lung rest strategy with a drastic decrease in driving pressure and PEEP while on VV-ECMO. The pathogenesis of this phenomenon occurs through shear stress, systemic inflammatory response syndrome (SIRS), and surfactant dysfunction following atelectasis-associated trauma (atelectrauma) [10]. Therefore, it may be crucial to maintain higher levels of PEEP (e.g., >10 cm H₂O) and decrease FiO₂ to limit atelectrauma and resorption atelectasis, respectively, decreasing the risk of complete or partial lung collapse.

One study found that after the initiation of VV- or VA-ECMO, there was an almost ubiquitous (>93%) worsening of pulmonary opacities on imaging in the study group [11]. Increasing PEEP and respiratory rate facilitates recruitment and has been shown to improve oxygenation and lung opacification [12].

Our case series focuses on the acute lung opacification or “white-out” that occurred within hours of initiating VV-ECMO in these patients. Previous studies have postulated that asymmetric lung compliance aggravates unilateral lung hyperinflation [13]. To our knowledge, we are the first to propose the hypothesis that asymmetrical airway compliance and resistance in COPD and asthma, in addition to unilateral hyperinflation, can also contribute to patchy areas of atelectasis, leading to unilateral lung collapse. Further prospective studies are warranted to explore the effects of asymmetrical lung compliance and resistance.

One proposed strategy to limit severe atelectasis upon ECMO cannulation involves maintaining PEEP in the range of 12-15 cm H₂O while targeting low tidal volumes (3-4 mL/kg) and a respiratory rate of 10/min. Once PaO₂ stabilizes on serial arterial blood gases (ABGs), FiO₂ should be aggressively weaned to less than 40% [14]. High levels of PEEP must be monitored carefully so as not to cause hemodynamic instability and alveolar overdistention, while adequate alveolar distention with the application of optimal PEEP can lower plateau and driving pressures through optimal recruitment [14].

Several studies have shown the ability to improve aeration and oxygenation through recruitment maneuvers, such as sustained, intermittent increases in plateau pressures, which increase end-expiratory lung volume and reduce intrapulmonary shunt [15]. Others have demonstrated utility in the gradual reduction of tidal volumes to as low as 2 mL/kg with a concomitant increase in PEEP up to 25 cm H₂O [16]. While such methods intuitively improve oxygenation, their use must be weighed against the risk of high tidal volumes and barotrauma in delicate lungs. There is no uniform ventilator management protocol for reducing the development of lung opacities while on VV-ECMO in the literature, and providers are tasked with employing some combination of these strategies to individually manage each patient based on their primary pathology. It is pivotal to tailor the ventilatory management of these patients, bearing these concepts in mind. Guidelines for mechanical ventilation during ECMO from the Extracorporeal Life Support Organization, as well as the ECMO conventional ventilator protocol settings for the recent EOLIA (ECMO to Rescue Lung Injury in Severe ARDS), can be utilized [17].

In all our patients, transthoracic echocardiography was normal, and the differential diagnoses for complete white-out lung, such as mucus plugging, large pleural effusions, unilateral pulmonary edema, and large hemothorax, were ruled out before the initiation of recruitment maneuvers. Thoracic ultrasound can also aid in differentiating between pathologies and ruling out the differential diagnoses.

## Conclusions

Given the increased utility of ECMO, the intensive care team must be aware of the ventilatory changes in patients with obstructive lung pathology and the possible complications associated with the lung rest strategy while on VV-ECMO, facilitating early recognition and prompt intervention. More research is needed to define parameters and ECMO indications in severe obstructive pulmonary diseases and mechanical ventilation strategies that minimize ventilator-induced lung injury.
